# Anlotinib as a promising inhibitor on tumor growth of oral squamous cell carcinoma through cell apoptosis and mitotic catastrophe

**DOI:** 10.1186/s12935-020-01721-x

**Published:** 2021-01-09

**Authors:** Zhaoming Deng, Wei Liao, Wei Wei, Guihua Zhong, Chao He, Hongbo Zhang, Qiaodan Liu, Xiwei Xu, Jun Liang, Zhigang Liu

**Affiliations:** 1 The Cancer Center of The Fifth Affiliated Hospital of Sun Yat-Sen University, Zhuhai, 519000 Guangdong China; 2 Department of Oral and Maxillofacial Surgery, The Fifth Affiliated Hospital of Sun Yat-sen University, Zhuhai, 519000 Guangdong China; 3 Guangdong Provincial Key Laboratory of Biomedical Imaging, The Fifth Affiliated Hospital, Sun Yat-sen University, Zhuhai, 519000 Guangdong China; 4 Department of Otolaryngology, The Fifth Affiliated Hospital, Sun Yat-sen University, Zhuhai, 519000 Guangdong China

**Keywords:** Oral squamous cell carcinoma, Anlotinib, VEGFR-2, Cell Apoptosis, Cell cycle arrest, Mitotic catastrophe

## Abstract

**Background:**

Oral squamous cell carcinoma (OSCC) has been one of the most malignant cancers in head and neck region. Anlotinib is a tyrosine kinase inhibitor targeting several receptors such as vascular endothelial growth factor receptor (VEGFR), fibroblast growth factor receptor (FGFR), platelet-derived growth factor receptor (PDGFR) and c-Kit. Here we investigated whether Anlotinib have any antitumor effect on oral cancer and tried to explore and explain the possible mechanism.

**Methods:**

Data from The Cancer Genome Atlas and the Gene Expression Omnibus and Gene Expression Omnibus database was collected to analyze the relationship between the expression of vascular epithelial growth factor receptor 2 and the overall survival rate of OSCC. Oral cancer cell lines Cal-27 and SCC-25 were cultured to conduct all the experiments. In vitro experiments such as CCK-8, colony formation, cell cycle assay and cell apoptosis assay were conducted to detect cell proliferation ability and the change of cell phase and apoptosis. Proteins concerning cell cycle and cell apoptosis were visualized via western blot. α-Tubulin were visualized via immunofluorescence to detect cells undergoing mitotic catastrophe.

**Results:**

Higher expression of VEGFR-2 was significantly related to poorer prognosis. Experiment in vitro demonstrated that cell proliferation was significantly inhibited(p < 0.05) after Anlotinib administration and G2/M arrest and apoptosis were both detected in both cell lines. Cycle-related proteins promoting cell cycle progression and proteins related to cell survival were downregulated in Anlotinib group compared to the control group. Cell-death-related biomarker and phosphorylated histone 3 were upregulated in expression in Anlotinib group. Abnormal spindle apparatus was observed in cells undergoing mitotic catastrophe.

**Conclusions:**

Anlotinib could exert an antitumor effect on oral cancer cell lines via apoptotic pathway and mitotic catastrophe pattern, presenting a promising potential therapy for patients with OSCC.

## Background

Oral squamous cell carcinoma has been one of the most malignant cancers in the head and neck region whose incidence is rising globally especially in regions or countries with heavy tobacco-consumption and alcohol abuse. Males could be affected by OSCC more often than females for much more frequent exposure to tobacco and alcoholic drinking [1]. In India, however, females present higher risks of intraoral carcinoma because of heavier indulgence of tobacco chewing behavior [2, 3]. It is reported that the incidence of the OSCC in recent decades is showing a significant climbing among younger patients in western countries [4]. Unfortunately, survival rate has not improved obviously in patients with OSCC. Though combination of surgical intervention and chemotherapy could remove the primary lesion in situ properly, survival rate and prognosis of patients with metastasis remains pretty low [5].

Anlotinib is a tyrosine kinase inhibitor targeting several receptors such as vascular endothelial growth factor receptor (VEGFR), fibroblast growth factor receptor (FGFR), platelet-derived growth factor receptor (PDGFR) and c-Kit. There are researches showing that Anlotinib has obvious antitumorigenic effect in several cancer such as thyroid cancer, soft tissue sarcoma and metastatic colorectal cancer [6–8]. In a phase-3 randomized clinical trial covering 439 patients with advanced non-small cell lung cancer, Anlotinib brought a prolonged overall survival rate and progression-free survival rate [9]. However, there is little study looing into the efficacy of the administration of Anlotinib on patients with OSCC. Here we investigated in vitro whether Anlotinib have any antitumor effect on oral cancer and tried to explore and explain the possible mechanism.

## Materials and methods

### Cell lines culture

Human oral squamous cell lines Cal-27 and SCC-25 were obtained from Genechem Co, Ltd (Genechem, Shanghai, China) and cultured carefully in RPMI 1640 Medium(Cal-27) and DMEM/F12(1:1) (SCC-25) (Thermo Fisher Scientific, MA, USA) consisting of 10% fetal bovine serum (FBS) (Thermo Fisher Scientific, MA, USA) and 1% penicillin/streptomycin (Thermo Fisher Scientific, MA, USA). Cell culture was maintained at 37 °C with 5% CO_2_ in a humidified incubator before the following in vitro experiments.

### The Cancer Genome Atlas (TCGA) and the Gene Expression Omnibus (GEO) data

Gene expression data from TCGA (https://tcga-data.nci.nih.gov/tcga/) and GEO (https://www.ncbi.nlm.nih.gov/geo/) was downloaded and collected to analyze the relationship between the prognosis of head and neck squamous cell carcinoma (HNSC) and VEGFR-2 expression.

### Cell proliferation viability assay

Cell counting kit 8 (CCK-8) (MedChemExpress, USA) was adopted to examine the cell proliferation viability. Cells were seeded in a 96-well plate with a density of 2000 cells per well. To find out an optimal toxicity effect on the cancer cells, Anlotinib with various concentration was added 24 h after seeding in the plate of each cell lines before CCK-8 (1:10 diluted in 100 µL medium per well) was added in each plate 24, 48 and 72 h later. After incubation of the mixture for 3 hours, measurement of Formazan generated was carried out at 450 nm using a microplate reader (Molecular Devices CMax Plus, USA).

### Colony formation assay

Cells were seeded in 6-well plates at a density of 300 cells per well. 24 h later, the medium was replaced by fresh one with Anlotinib of the optimal concentration resulted from CCK-8 Assay while medium of control group was replaced without any change in constitution. After another 24 h of incubation together, medium of both groups was removed to be added with fresh Anlotinib-free medium, followed by 14-day incubation during which medium was freshened on a regular basis. At the end of the assay, colonies were visible enough to be fixed with 4% Paraformaldehyde (Bioss, Beijing, China) for 15 min and another 15-min staining with crystal violet (Sigma Life Science), after which the results were all processed with the help of ImageJ.

### Cell cycle and cell apoptotic assay

Cells were incubated with or without Anlotinib for 24 h and harvested before staining with Propidium iodide (PI, BD Biosciences, USA). To detect apoptotic percentage, cells of different groups were harvested to be stained with FITC (BD Biosciences, USA) and Annexin-V (BD Biosciences, USA) before the flow cytometry analysis (DeFlex, Beckman Coulter, Inc.USA). Data from cell cycle was transformed to graphs with the help of ModFit LT3.0 (VerifySoftware House, Topsham, ME, USA) while apoptotic data was visualized via FlowJo V10.0(BD Biosciences, USA).

### Western blotting

Western blotting was performed to detect proteins related to cell cycle and cell apoptosis. Antibodies were used to detect proteins including cdc2 (#9116, 1:1000, CST), phospho-cdc2 (#4539, 1:1000, CST), Chk2 (#6334, 1:1000, CST), phospho-Chk2 (#2197, 1:1000, CST), CyclinB1 (#12231, 1:1000, CST), Bax (#5023, 1:1000, CST), Bcl-2 (#15,071, 1:1000, CST), Caspase-3 (#14,220, 1:1000, CST), Cleaved Caspase-3  (#9664, 1:1000, CST), PARP (#9532, 1:1000, CST), Cleaved PARP (#5625, 1:1000, CST), phospho-Histone H3 (#53,348, 1:1000, CST) and α-tubulin (#2125, 1:1000, CST). β-Actin was chosen as internal control. Signal was visualized via Supersignal Western Blot Enhancer (#46,640, Thermo Fisher Scientific, USA) using a Chemiluminescent Imaging System (Tanon, Shanghai, China).

### Immunofluorescence assay

Immunofluorescence assay was performed in a 24-well plate for both cell lines. Cells were harvested and fixed with 4% paraformaldehyde and permeated with 0.3% Triton X-100. After blocking with 5% BSA (Bovine Serum Albumin), cells were incubated overnight at 4 ℃ with primary antibodies α-tubulin (#2125, 1:25, CST). Fluorophore-conjugated secondary antibodies (#4414, 1:400, CST) were used to incubate the specimens at room temperature for 1 h. DAPI was used to stain the nuclear of the cells in the final step. All of the steps were operated in the dark after adding Fluorophore-conjugated secondary antibodies. Specimens were finally observed and images were captured under an immunofluorescence microscope. In each image, apoptotic cells with breaking karyotheca were considered to be undergoing mitotic catastrophe and counted. Percentage of nuclear-breaking cells was calculated and compared between groups.

### Statistical analysis

All the experiments were carried out three times and all the data collected was analyzed by SPSS 17.0 (SPSS, Inc., Chicago, IL, USA). Survival curves were drawn using Kaplan–Meier method. Log-rank test was adopted to compare high and low expression groups for VEGFR-2. Two-tailed P value less than 0.05 was defined as statistically significant.

## Results

### Poor prognosis was related to higher expression of VEGFR-2

**Fig. 1:**
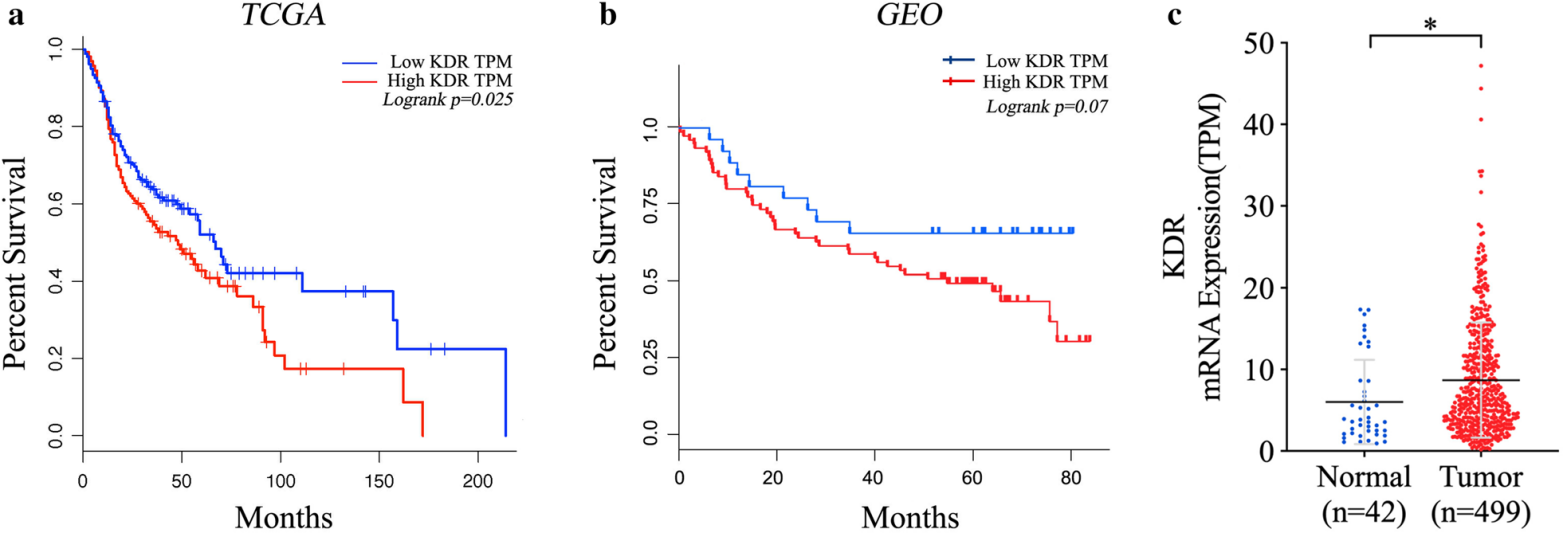
Data from TCGA and GEO databases were collected to analyze the relationship between prognosis and the expression of VEGFR-2. TCGA data concerning 517 specimens showed poor prognosis were closely related to high expression of VEGFR-2 (p < 0.05) (**a**) while data from GEO demonstrated slight relationship (p = 0.07) (**b**). Higher expression of mRNA was found in tumoral tissues (n = 42) than in adjacent normal tissues (n = 499) when analyzing data from TCGA database. (*p < 0.05, **p < 0.01)

As VEGFR-2 was the major target of Anlotinib [10], data covering 517 specimens (259 high expression and 258 low expression) from TCGA demonstrated that poor prognosis was significantly related to higher expression of VEGFR-2 (p < 0.05) (Fig. 1a). Data concerning 93 samples from the Gene Expression Omnibus presented a slight relationship between the overall survival rate and high expression of VEGFR-2 (p = 0.07) (Fig. 1b). Expression was found to be significantly higher in tumoral tissues than in the adjacent normal tissues (p < 0.05) (Fig. 1c).

### Anlotinib suppressed short and long-term proliferation of oral cancer cell lines

**Fig. 2:**
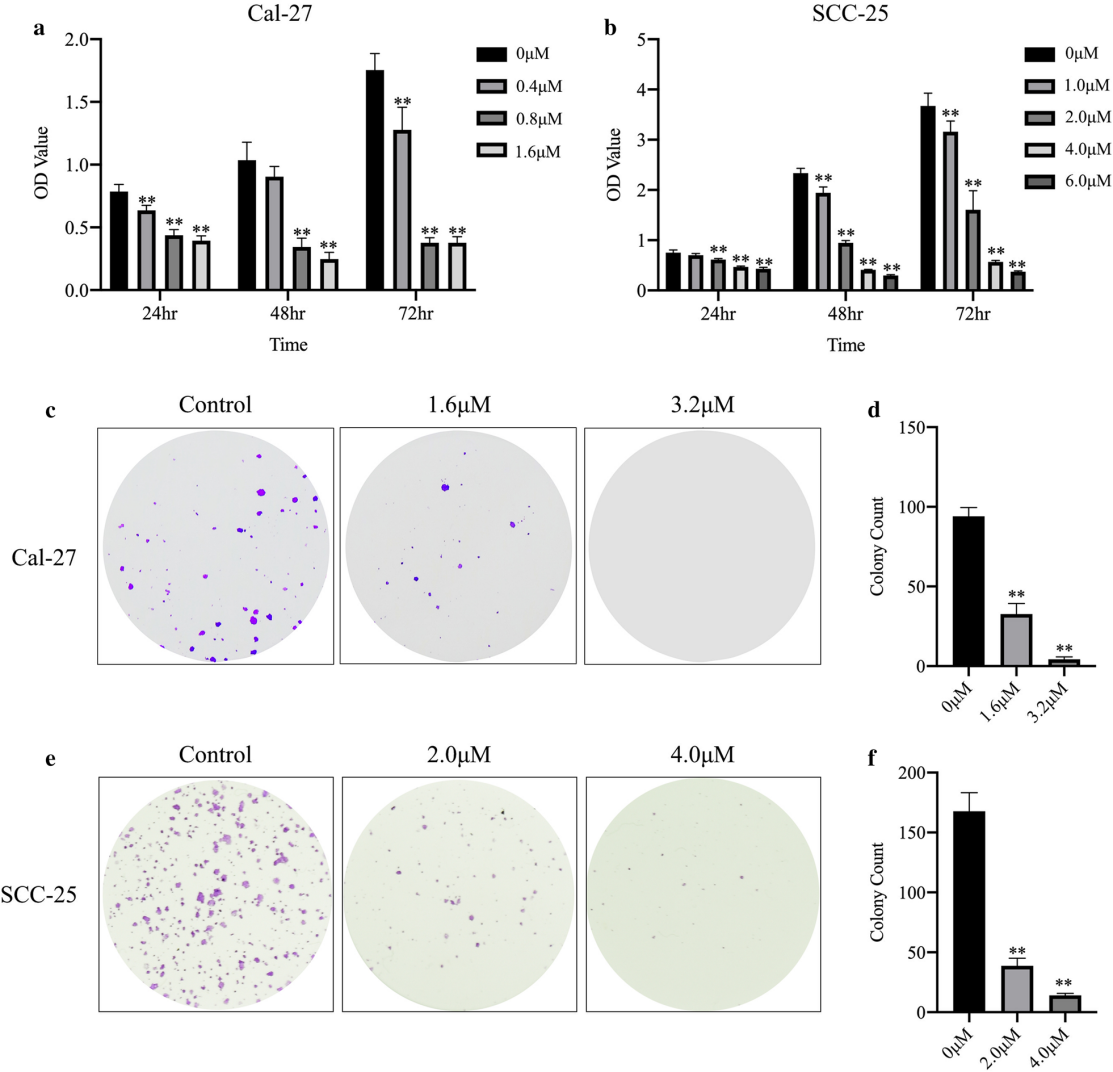
Anlotinib inhibited cell viability of proliferation. Three consecutive days of CCK-8 test showed Anlotinib suppressed cell proliferation in a dose-dependent pattern (**a**, **b**). Colony formation confirmed the inhibitive effect of Anlotinib. (**c–f**)(*p < 0.05, **p < 0.01)

The results of CCK-8 assay demonstrated that cell proliferation in short term of oral cancer cell lines (Cal-27 and SCC-25) was significantly suppressed by Anlotinib with a dose-dependent pattern (p < 0.01) (Fig. 2a, b). Colony formation was performed for each cell line to find that long-term cell proliferation was greatly inhibited by Anlotinib compared to the control group (p < 0.01). (Fig. 2c–f)

### Cell cycle arrest and cell apoptosis were induced by Anlotinib

**Fig. 3:**
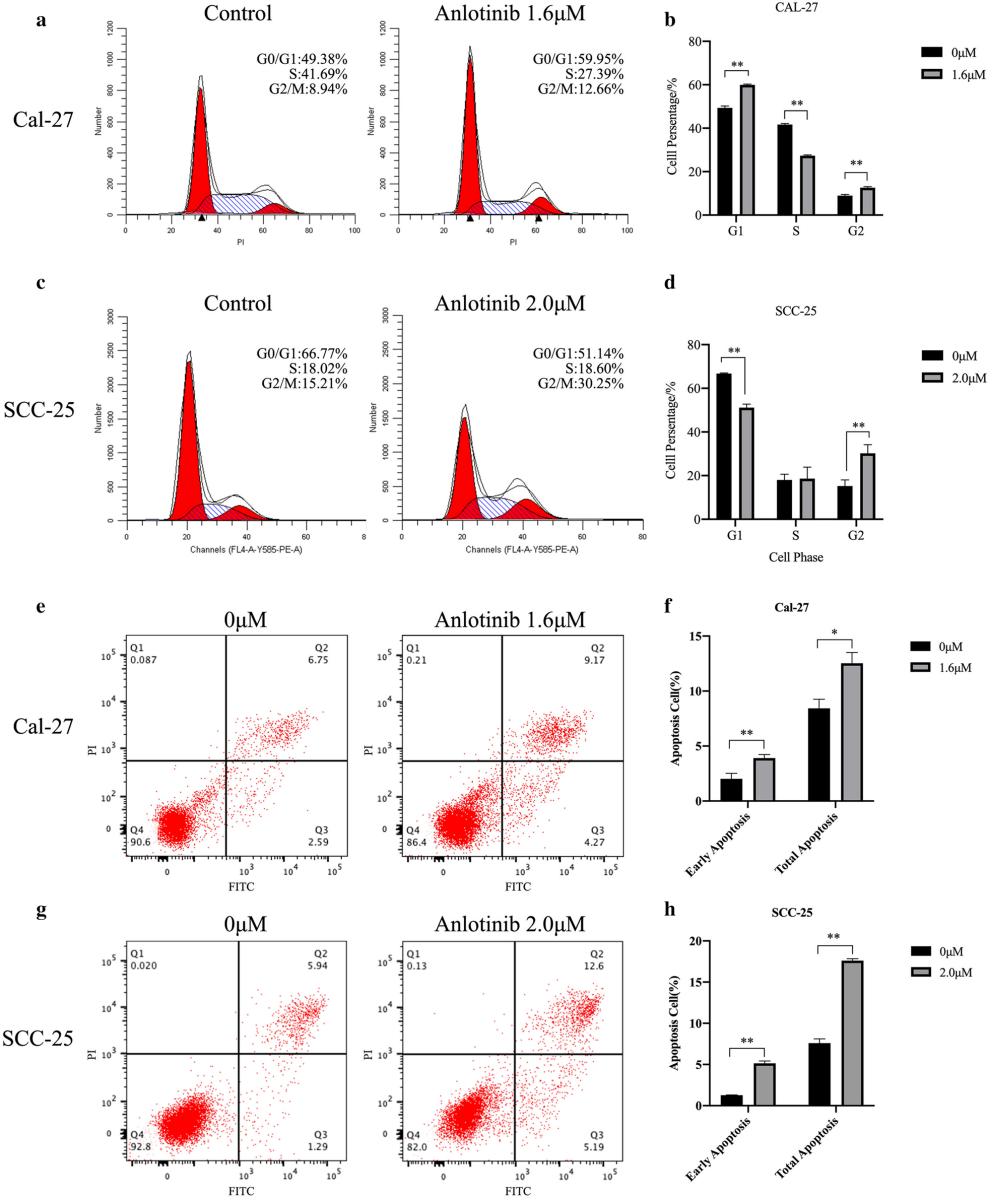
Cell cycle arrest and cell apoptosis were induced by Anlotinib. Cells were treated with Anlotinib for 24 h before harvest for flow cytometry to detect cell cycle distribution, and for 48 h to detect cell apoptotic percentage. The results demonstrated cell cycle percentage of G2 was significantly raised from 8.94% (Cal-27) and 15.21% (SCC-25) to 12.66% and 30.25% (**a**–**d**). Cell apoptotic percentage (early apoptosis and total apoptosis) was significantly increased in groups treated with Anlotinib (**e**–**h**). (*p < 0.05, **p < 0.01)

G1/S and G2/M arrest were detected in cell line Cal-27 treated with Anlotinib. (p < 0.01) (Fig. 3a, b) while G2/M arrest was observed in SCC-25 treated with Anlotinib. (p < 0.01) (Fig. 3c, d). Compared to the control group, cell apoptosis, including early apoptosis and general apoptosis, was induced in both cell lines treated with Anlotinib. (P < 0.05) (Fig. 3e–g).

### Down-regulated expression of VEGFR-2 was induced by Anlotinib

**Fig. 4:**
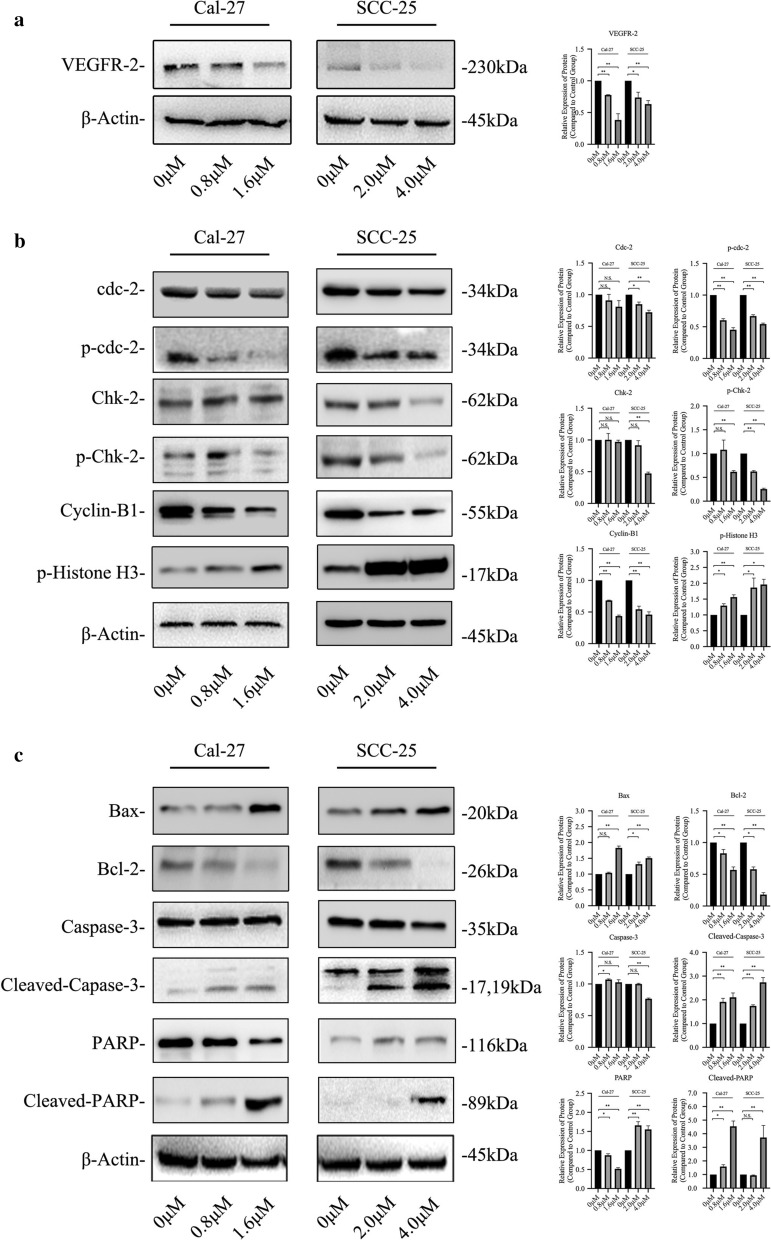
Expression of VEGFR2, cell-cycle-related and apoptosis-related protein was visualized by western blot. Anlotinib inhibited the expression of VEGFR2 on cell line Cal-27 and SCC-25 (**a**). Key checkpoint biomarkers such as phosphorylated-Chk-2, phosphorylated-Cdc-2 and Cyclin B1 was down-regulated after Anlotininb administration. Phosphorylated histone 3 was upregulated in anlotinib-treated group (**b**). Expression of such apoptotic biomarkers as Bax, Cleaved-Caspase-3 and Cleaved-PARP increased in Anlotinib-treated groups. Bcl-2 was downregulated in expression (**c**)

Western blot was conducted to find that the expression of VEGFR-2 was decreased in both cell lines treated by Anlotinib in a dose-dependent pattern (Fig. 4a).

### Cell cycle procedure was interrupted by Anlotinib

Expression of cell-cycle-related proteins including cdc-2, phosphorylated cdc-2, Chk-2, phosphorylated Chk-2 and Cyclin B1, was detected via western blot (Fig. 4b). Obviously, expression of phosphorylated cdc-2 and Chk-2 was downregulated in both cell lines after Anlotinib administration. Cyclin B1 was also decreased in expression. Phosphorylated histone 3 was detected to increase in expression.

### Cell death was induced by the way of cell apoptosis with change in expression of related proteins

Survival-related biomarker Bcl-2 was detected to be inhibited while proteins concerning cell apoptosis such as Bax, Caspase-3(phosphorylated or non-phosphorylated) and PAPR (phosphorylated or non-phosphorylated) were found to be upregulated after Anlotinib treatment (Fig. 4c).

### Nuclear instability and abnormal mitosis were induced as a result of Anlotinib administration

**Fig. 5:**
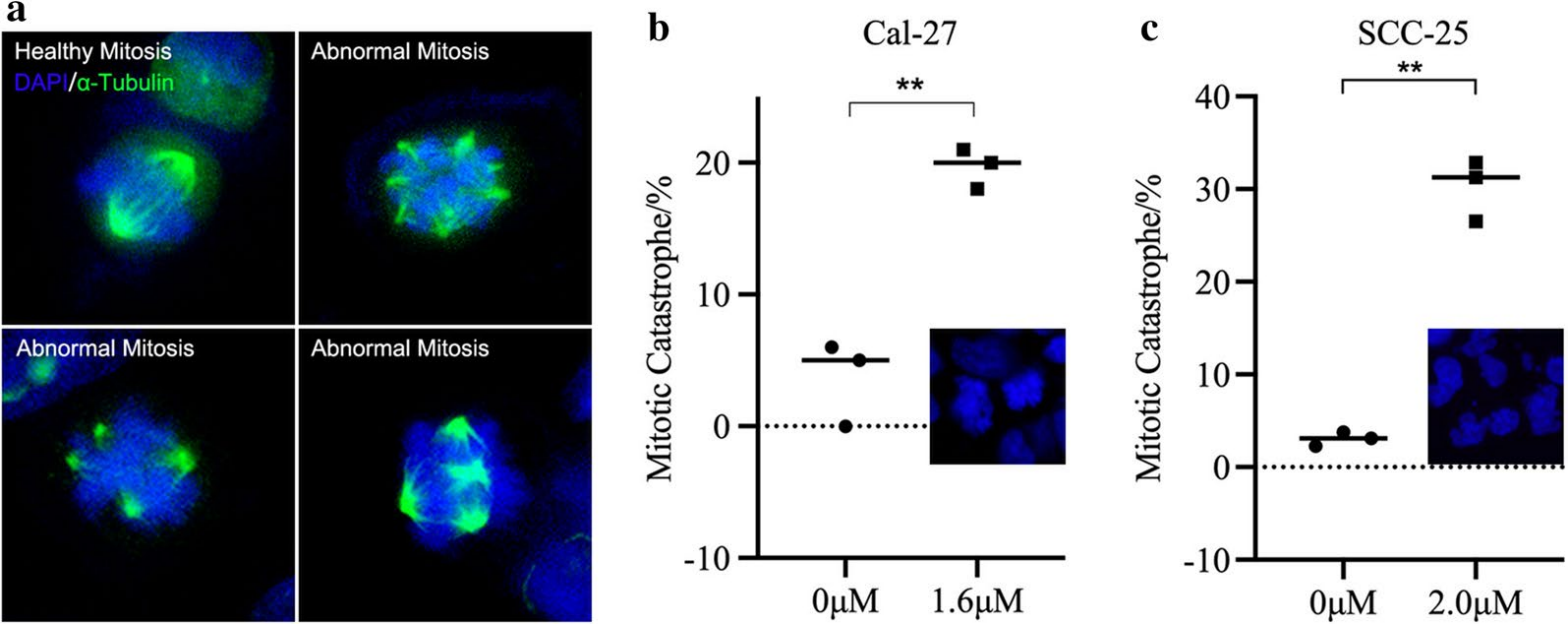
Nucleus chromosome instability and mitotic catastrophe were observed and visualized via immunofluorescence. Normal mitosis was presented in a bipolar pattern of spindles apparatus by α-Tubulin-labeled staining of immunofluorescence while in Anlotinib-treated group, spindles in cells were not heading to two but four poles or even more (**a**). Significant nuclear breakage was found in Anlotinib-treated group(20.1% and 30.21%) than in control group (5.4% and 3.043%) (*p < 0.05, **p < 0.01)

Abnormal spindle apparatus labeled with α-Tubulin was captured in Anlotinib group of Cal-27 and SCC-25, which was characterized with tetraploid and poly-central mitosis (Fig. 5a). Higher percentage of nuclear breakage was found in Anlotinib group than control group (Fig. 5b, c).

## Discussion

As a multitargeting tyrosine inhibitor in clinical development, Anlotinib exerts remarkable antitumor effects through anti-angiogenesis activity targeting multiple tyrosine kinases such as vascular endothelial growth factor receptor 1/2/3 (VEGFR-1/2/3), platelet-derived growth factor receptor (PDGFR) and fibroblast growth factor receptor (FGFR) [10]. It was reported that Anlotinib have significant antitumoral effect on solid tumor including thyroid cancer [11], non-small lung cancer [12], soft tissue sarcoma [13], renal cancer [14] and gastric cancer [15]. However, there is little research focusing on whether Anlotinib have an antitumor effect on oral squamous cell carcinoma which is one of the most common cancers in head and neck region.

The regulation of cell cycle involves complicated molecular activities and changes in protein level. Cyclin-dependent kinase 1(CDK1), also called cell division control protein 2 (Cdc2), is fundamental for cells to transform from G2 phase to mitotic phase [16]. WEE1 and Chk1 are key checkpoint kinases regulating the activity of cdc2, guaranteeing a healthy mitosis progression and prevent cells from entering mitosis with incomplete or damaged DNA replication [17–19]. Cdc2 remains inactive till G2 phase when the concentration of cyclin B reached high, allowing the formation of Cdc2-cyclin B. With the help of cdc25c phosphatases removing the cdc2 inhibitory phosphorylation sites Thr 14 and Tyr15, cdc2-Cyclin B complex turn highly active during mitotic entry. Chk1 and Chk2 are essential checkpoints of gene stability and integrity, which regulate basic cellular activities such as DNA replication, cycle progression and chromosome remodeling [20]. There are researches focusing on Chk1 and Chk2 knockout and knockdown models whose results showed the essential role Chk1 played in G2/M transition and Chk2 in cell apoptosis as well as S phase checkpoint regulation [21, 22]. Besides, increasing studies have discovered the overlapping phenomenon of Chk1 and Chk2 in cell cycle progression and checkpoint signaling, indicating the closely-related relationship with cell cycle and apoptosis [23, 24].

Cell death was considered to include several forms such as apoptosis (Programmed cell death), necrosis (Uncontrolled cell death), autophagy, ferroptosis, oncosis, pyroptosis and mitotic failure [25–27]. Pathway of cell death influences the extent and selection of modification in gene and protein level [28]. Mitotic catastrophe is a one of the cell death pathways that characterizes several abnormal cellular biological processes such as defect of nuclear division, multinucleation or micronucleation, which could be a result of factors including DNA damage, abnormal checkpoint of cell cycle especially the M phase and spindle apparatus, microtubes poisoning, overheat and presence of tetraploid. A healthy G2/M transformation is greatly dependent on an undisrupted progress of the combination of cyclin-dependent kinase 1 (CDK-1) and cyclin B1 which guarantees multiple important nuclear activities such as the condensation of chromosome, breakdown of nuclear membrane during mitosis and remodeling of microtubes [29]. Anaphase-promoting complex (APC), a key driver of the degradation of cyclin B and securin during middle-late phase, the end of mitosis, could disturb normal progression of cell cycle and induce mitotic failure by prolonging activation period of CDK-1 as a result of a consistent self-inhibition [30].

In our work, we found a downregulation of phosphorylated cdc-2 (p-cdc-2) and a decreasing Cyclin B1 after Anlotinib administration, which significantly perturbed the normal transformation of cell trying to start up mitosis. Failure of combination of p-cdc-2 and Cyclin B1 during the late stage of cell cycle is a depending factor to induce G2/M arrest. Blockage of cell cycle along with generation of apoptotic signals disrupted microtubes condensation and chromosome instability. Histone within nuclear plays a key role in regulating gene expression [31]. Different kind of modification of histone have different effect on gene regulation. Generally, phosphorylated form of histone could bother the normal tie with DNA which consequently destabilizes the chromosome and disturbs the remodeling and condensing of homologous chromosome during mitosis [31]. In our work, an upregulated expression of phosphorylated Histone 3 was detected via western blot and immunofluorescence, indicating cells were undergoing chromosome disturbance. α-Tubulin was visualized to monitor the contour of spindles apparatus in mitotic cell via immunofluorescence. Unlike healthy mitosis featuring bipolar pattern, abnormal spindles were observed in cells after Anlotinib administration, characterizing the presence of tetraploid or even multipolar spindles which doomed a death for a cell.

## Conclusions

Within the limitation of the research, Anlotinib exerted an antitumor effect on oral squamous cell carcinoma cell lines (Ca-27 and SCC-25) by inducing cell death via cell apoptosis and cell cycle arrest. Disorder of cycle checkpoints combined with generation of apoptotic signals might be a reason to induce abnormal mitosis. Failure of condensation of spindle apparatus was a key driver to induce mitotic catastrophe, rendering chromosome instability and a nuclear or cell configuration far from normal pattern. Significant antitumor effect was observed in vitro experiments, indicating that Anlotinib might have a potential curable effect on patients with OSCC with a hopeful efficacy.

## Data Availability

All data generated or analyzed during this study are included in this published article and its additional files.

## Abbreviations

OSCC: Oral squamous cell carcinoma
VEGFR: Vascular endothelial growth factor receptor
FGFR: Fibroblast growth factor receptor
PDGFR: Platelet-derived growth factor receptor
CCK-8: Cell counting kit-8
TCGA: The Cancer Genome Atlas
GEO: The Gene Expression Omnibus
HNSC: Head and neck squamous cell carcinoma
Cdk-1: Cyclin-dependent kinase 1
APC: Anaphase-promoting complex
